# *mcstas_gisans*: combining ray tracing with the distorted-wave Born approximation using *McStas* and *BornAgain* for virtual GISANS experiments

**DOI:** 10.1107/S160057672600213X

**Published:** 2026-04-22

**Authors:** Milán Klausz, Artur Glavic, Sebastian Köhler, Thomas Arnold, Nicolò Paracini, Philipp Gutfreund, Marité Cárdenas, Max Wolff, Tommy Nylander

**Affiliations:** a Institute for Energy Security and Environmental Safety HUN-REN Centre for Energy Research Konkoly Thege Miklós út 29-33 1121 Budapest Hungary; b European Spallation Source ESS ERIC PO Box 176 SE-221 00 Lund Sweden; c PSI Center for Neutron and Muon Sciences, 5232 Villigen, Switzerland; d Physical Chemistry, Lund University, Lund, Sweden; e Institut Laue–Langevin, 71 avenue des Martyrs, CS 20156, 38042 Grenoble Cedex 9, France; f Department for Biomedical Science and Biofilms – Research Center for Biointerfaces, Faculty of Health and Society, Malmö University, 205 06 Malmö, Sweden; g Instituto Biofisika (UPV-EHU/CSIC) Fundación Biofisika Bizkaia 48940 Leioa Spain; h Ikerbasque Basque Foundation for Science 48013 Bilbao Spain; i Department for Physics and Astronomy, Uppsala University, Box 512, 75120 Uppsala, Sweden; j NanoLund, Lund University, Lund, Sweden; k LINXS Institute of Advanced Neutron and X-ray Science, Lund, Sweden; l School of Chemical Engineering and Translational Nanobioscience Research Center, Sungkyunkwan University, Suwon 16419, Republic of Korea; Lund University, Sweden

**Keywords:** grazing-incidence small-angle neutron scattering, GISANS, instrumentation, scientific computing, *mcstas_gisans*, *McStas*, *BornAgain*

## Abstract

The *mcstas_gisans* framework is a collection of Python scripts and modules to facilitate the simulation of grazing-incidence small-angle neutron scattering (GISANS) experiments. This approach combines *McStas* instrument simulation with *BornAgain* sample modeling capabilities. The Monte Carlo Particle Lists format for particle trajectory allows exchange between simulations that enables seamless transition from instrument modeling to sample scattering analysis. The Python-based processing utilities handle data transformation, scaling to virtual experiment times for absolute intensities, and visualization. The required software environment is managed through Conda, ensuring reproducible deployments across platforms. This integrated approach facilitates accurate simulation and analysis and enables the comparison of the GISANS capability of different neutron scattering instruments.

## Abstract 2

*mcstas_gisans* is a Python-based framework that enables simulation of grazing-incidence small-angle neutron scattering experiments by integrating *McStas* and *BornAgain* into a single workflow.

## 1. Introduction

The increasing interest in studying interfacial structures in such diverse applications as solid state materials, magnetism, soft matter and life sciences calls for further exploitation and development of surface techniques. Traditional surface studies with X-rays or neutrons involve measurements of the specular reflectivity (Penfold & Thomas, 1990 ▸). This allows the extraction of information about the density profile of materials perpendicular to the plane of an interface in various areas of science, including the study of solid–liquid interfaces (Wolff *et al.*, 2024 ▸), coatings (Wolff & Gutfreund, 2021 ▸) and magnetic thin films (Toperverg & Zabel, 2015 ▸; Gayen *et al.*, 2017 ▸). Additional information on the lateral correlations in the plane of the interface can be obtained by studying the off-specular grazing-incidence and/or near-surface scattering (Wolff, 2018 ▸). At small incidence angles of the incoming neutron beam, close to the critical angles of total external reflection at the substrate interface, the measured grazing-incidence scattering is surface sensitive and can probe features in the surface layer (*x* and *y* interface plane, see Fig. 2) down to sub-nanometres. The X-ray version, grazing-incidence small-angle X-ray scattering (GISAXS), has grown rapidly over recent years. There are today many synchrotron beamlines offering the technique, as well as an increasing number of laboratory sources capable of measuring in this mode. The widespread usage of GISAXS is possible due to the very intense and inherently brilliant X-ray sources and the simple beamline geometry required to perform these measurements. Grazing-incidence small-angle neutron scattering (GISANS) (Müller-Buschbaum, 2016 ▸; Hamilton *et al.*, 1994 ▸) is a more challenging technique because of the lower brilliance of neutron sources and higher propensity for incoherent scattering adding to the background. For the typically small sample volumes used for many studies, this results in a significantly lower signal-to-noise ratio. However, the technique offers several significant advantages over GISAXS. Isotopic substitution, which improves sensitivity to specific components within a system, and the penetrating power of neutrons allow access to buried interfaces that are inaccessible with X-rays. Here, neutrons are particularly sensitive to light elements (most importantly hydrogen), which is a significant advantage for soft matter and life science, as well as energy storage and conversion applications. Furthermore, the sensitivity to magnetic induction allows the study of quantum materials, such as magnetic thin films.

The GISANS technique combines two well established methods, namely neutron reflectometry (NR) and small-angle neutron scattering (SANS). As a consequence, GISANS experiments are often performed on instruments that are optimized for either transmission SANS or NR and, consequently, are not optimized for GISANS. Explicitly, reflectometers commonly use relaxed divergence in the plane of the sample interface, in order to improve flux but resulting in relatively poor resolution in the plane of the interface. On the other hand, SANS instruments usually do not have appropriate collimation or sample-alignment capacity optimized for grazing-incidence geometry. Furthermore, the optimization of wavelength bandwidth and resolution required for GISANS as opposed to SANS or NR is quite different, as GISANS requires high resolution to enable depth sensitivity, while SANS often uses relaxed resolution, in particular with respect to wavelength.

The analysis of grazing-incidence small-angle scattering experiments has developed considerably, with software packages such as *BornAgain* (Pospelov *et al.*, 2020 ▸) now offering the ability to conveniently perform simulations using the distorted-wave Born approximation (DWBA) for a wide variety of sample types. Still, such analysis is challenging as experiments are often performed on instruments in an unusual configuration. It can be difficult to disentangle whether the limitations in performance and data quality originate from the sample or the instrument. One way to improve both the post-experiment analysis and the pre-experiment instrument setup is to include the instrument directly in simulations. This would have a number of benefits, including (i) optimized beamline settings for GISANS, (ii) the ability to benchmark new beamline concepts for GISANS against existing SANS and NR beamlines as well as against each other, and (iii) better control over instrument parameters to facilitate the data analysis.

*McStas* is a widely used Monte Carlo simulation tool dedicated to model neutron scattering instruments (Lefmann & Nielsen, 1999 ▸; Willendrup & Lefmann, 2020 ▸). Its component-based architecture enables modeling of complex instrument configurations, predicting performance under various conditions, to support decision making in both instrumentation and experiment planning. Its wide library of components and instruments, partly provided by the user community, greatly reduces the effort needed for simulation projects (Willendrup & Lefmann, 2021 ▸).

Although a wide range of samples are available in *McStas*, there is no component that offers GISANS simulations with a flexible sample definition option in the way that *BornAgain* does. Therefore, we have created a connection between *McStas* and *BornAgain*, in a similar way to what has been done between other simulation tools (Klausz *et al.*, 2021 ▸; Kanaki *et al.*, 2018 ▸; Stefanescu *et al.*, 2019 ▸). Creating such a connection can be facilitated by Monte Carlo Particle Lists (MCPL) (Kittelmann *et al.*, 2017 ▸), a binary format dedicated to interchanging particles between various Monte Carlo simulation applications. Converters and plugins for several simulation packages already exist, but the transition to *BornAgain* requires additional coding effort.

Here, we present a framework, *mcstas_gisans*, designed to connect the *McStas* simulation of neutron scattering instruments with the *BornAgain* GISANS simulation of samples. The primary goal is to facilitate the comparison of GISANS instrument capabilities, thus enabling and supporting decision making for the design and setup of this type of instrumentation. Benchmarking of the framework is achieved by comparing simulation results with experimental data measured at the D22 instrument (Institut Laue–Langevin, ILL).

## 2. Method

The *mcstas_gisans* framework is a bridge between *McStas* and *BornAgain*. It consists of a collection of Python scripts and modules facilitating a workflow in which *McStas* and *BornAgain* are used, without being a self-contained application. The workflow involves the following three steps: (1) *McStas* simulation of the instrument up until the sample position, (2) *BornAgain* GISANS simulation of the sample, and (3) data processing and visualization.

The first step is a traditional *McStas* simulation with a few considerations regarding the instrument file to create the output files necessary for the subsequent step. The other two steps have their own dedicated Python scripts with command-line interfaces for user control. The sample simulation script uses the MCPL Python module to read the output of the *McStas* simulation and the *BornAgain* Python application programming interface (API) to run the *BornAgain* simulations. The data-processing script can be used to read the output of the sample simulation script, perform data treatment and visualize the results. The clear separation of these steps with intermediate files allows the reuse of results of computationally heavy simulations, eliminating the need to rerun them in the event of downstream changes.

### 2.1. *McStas* simulation

The *McStas* simulation is a standalone step in the workflow without any additional tool necessary other than a working installation of any *McStas* version. The only required modification for an existing *McStas* instrument model is the addition of an MCPL_output (MCPL Project, 2025 ▸) *McStas* component at the sample position. This component will save the state variables for all neutron rays from the previous component, with coordinates relative to the MCPL_output component itself. This MCPL file is the source of neutrons for the *BornAgain* simulation of the interaction with the sample in the next step of the workflow. *McStas* does not simulate individual physical neutrons. Instead, it simulates statistical representatives of them. This is reflected in the statistical weight assigned to each simulated neutron, which can be greater or less than one. For this reason, these simulated particles are often referred to as neutron rays or neutron events. Therefore, the neutrons stored in an MCPL file can represent either many physical neutrons sharing the same properties or just a fractional contribution of a single neutron.

Optionally, extra TOFLambda_monitor*McStas* components describing the time-of-flight (TOF)–wavelength distributions at certain parts of the instrument can be added. The output of these monitors can enable simulation options in the following steps, such as the later discussed TOF-based filtering, *t*_0_ correction and wavelength frame multiplication (WFM) mode (Löhmann *et al.*, 2020 ▸).

### 2.2. *BornAgain* simulation

The *BornAgain* simulation script is the core of the project, handling the interaction of neutrons with the sample and calculation of scattering-vector magnitude (*Q*) values. This is not a regular simulation script that can be exported from the *BornAgain* GUI. The progression of the script can be broken into the following steps: loading neutron ray parameters from an MCPL file, preconditioning the neutrons, processing neutrons through *BornAgain* simulations to generate *Q* values, and saving the results in a format that can be used for further processing and plotting in the subsequent data-processing and visualization step.

#### 2.2.1. Loading neutron parameters

**Figure 1:**
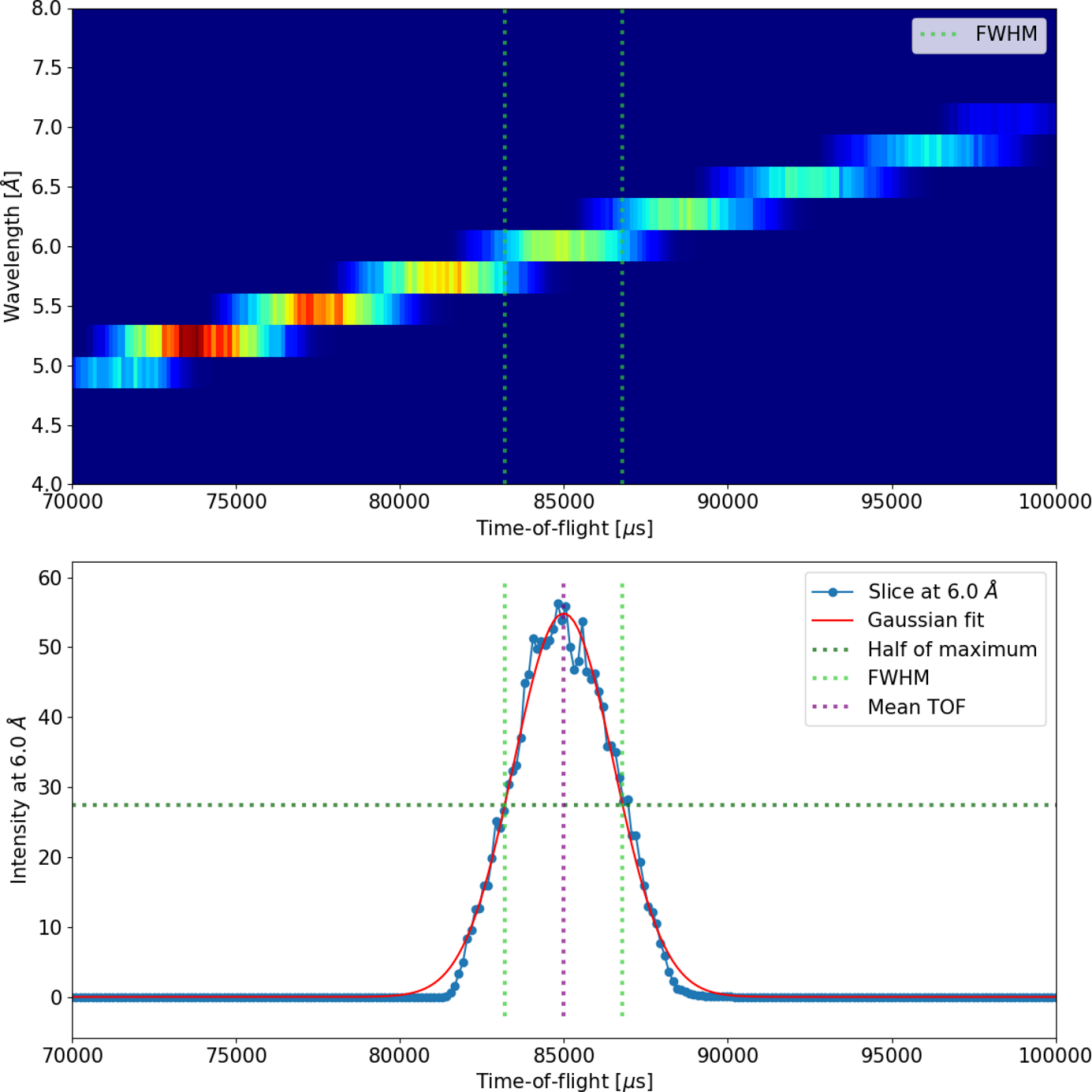
Demonstration of defining TOF filtering limits automatically on the basis of a TOF–wavelength *McStas* monitor (top) and a wavelength of interest (6.0 Å). The relevant wavelength bin containing the wavelength of interest is retrieved and fitted by a Gaussian function to find the FWHM limits (bottom).

Neutrons are loaded from an MCPL file created in a preceding *McStas* simulation. For a non-TOF instrument, a certain incident neutron wavelength range is selected by the monochromator in accordance with the simulated instrument settings; therefore, all neutrons in the file should contribute to the same result. However, for TOF instruments, a broad range of incident neutron wavelengths is probed within a single instrument setting, and data must be sliced in TOF – and thus in wavelength – to yield meaningful results. This means that in TOF data reduction the *Q* values are calculated for each detection event according to their TOF value and detection position, but then *Q* values are only integrated over certain TOF ranges, resulting in multiple 2D intensities corresponding to respective wavelength ranges (Müller-Buschbaum, 2012 ▸). In simulations, this could be done in the same way. However, in order to lower the computational resources needed for a simulation run, the current implementation aims to acquire the result only for a single TOF/wavelength slice. Of course, deciding which TOF slice the neutrons contribute to can only be correctly done on the basis of their TOF value at the detector, and to know that would require doing the full simulation of all neutrons. As an approximation, though, the TOF at the sample position can be used, which is already known from the *McStas* simulation. Only neutrons from an appropriate TOF range at the sample position are simulated using TOF limits within which they are expected to contribute to the same TOF/wavelength slice. The contributions from these different TOF ranges can then be added up in the same way as done for non-TOF instruments. In practice, this is achieved by filtering neutrons read from the MCPL file according to their TOF. Either the TOF limits can be set by hand, enabling users to set their own limits, possibly based on chopper calculations, or they can be defined automatically via a TOF–wavelength *McStas* monitor, describing neutrons at the sample position, and a selected wavelength of interest. In the latter case, TOF limits are determined as the full width at half-maximum (FWHM) limits from a Gaussian function fitted to TOF distribution of the relevant wavelength range, as demonstrated in Fig. 1 ▸. The accepted TOF range around the mean value can be modified by a multiplier value, and the reliability of the Gaussian fitting can be increased by rebinning along the wavelength axis by a provided factor, which can be important for low statistics or fine binning. Although the exact wavelength of each neutron is known from the *McStas* simulation, and filtering could, in principle, be performed directly on the basis of wavelength, it is essential to apply filtering in TOF. In experimental data reduction, the wavelength is always derived from the measured TOF, and each instrument as well as each specific instrument configuration has its own finite TOF resolution that translates into a corresponding wavelength resolution. Using TOF-based filtering in the simulation ensures that the wavelength distribution and its effect on the resulting *Q* resolution reflects the experimental conditions, leading to results that are directly comparable with measured data.

Although *McStas* is capable of high-fidelity simulations, as demonstrated for instruments with a simple design and a small number of components (Potashnikov *et al.*, 2024 ▸), user-defined simulation models are often not elaborate enough to reproduce absolute intensities. As a consequence, the simulated total intensity at the sample position – that is, at the end of the *McStas* simulation step – may differ from experimental expectations. To account for this, a uniform scaling factor is applied to the statistical weight of all neutrons before the scattering calculations. Uniform scaling of all neutron weights results in a simple modification of the absolute intensity without altering relative properties of the beam; therefore, it leads to a uniform scaling of the final results. The most straightforward way to determine a suitable scaling factor is by comparing the measured and simulated monitor intensities directly before the sample position. If no measured monitor data are available, the factor can instead be obtained by comparing the measured and simulated direct-beam intensities.

#### 2.2.2. Preconditioning the neutrons

Preconditioning of neutrons before the *BornAgain* simulation is necessary to bridge certain differences between *McStas* and *BornAgain*. This process includes a coordinate transformation and propagation to the sample surface. In addition, an optional *t*_0_ correction can be applied to adjust the TOF values of neutrons so that they correspond to the correct flight distance and pulse structure, ensuring consistency in data reduction.

**Figure 2:**
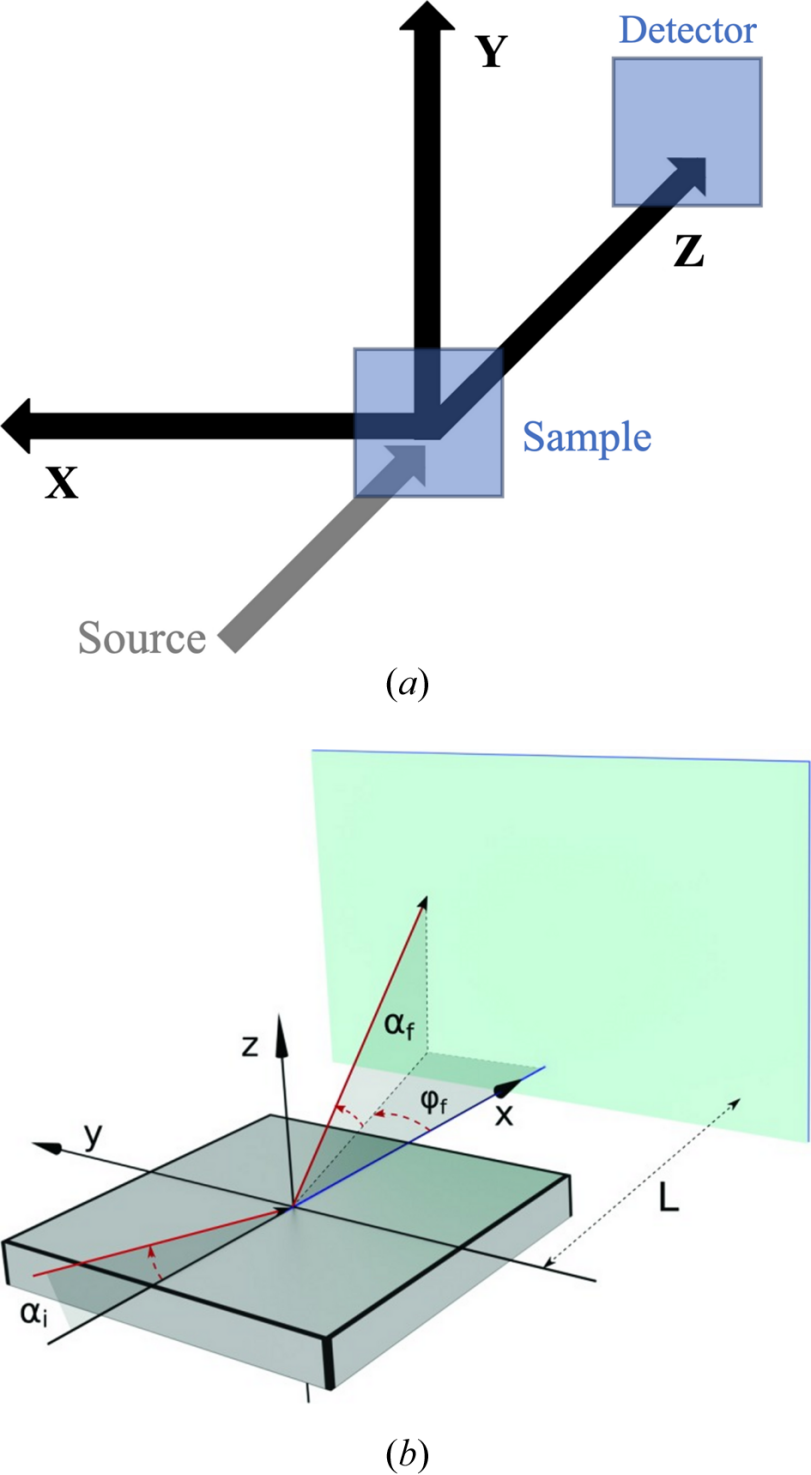
Difference of the coordinate systems: (*a*) the coordinate system used in *McStas* (Willendrup *et al.*, 2024 ▸) and (*b*) the geometric conventions in *BornAgain* (Pospelov *et al.*, 2020 ▸). Reproduced with permission of the International Union of Crystallography.

The coordinate transformation is necessary because there are fundamental differences in the geometric conventions of *McStas* and *BornAgain*, as demonstrated in Fig. 2 ▸. In *McStas*, the *z* axis points in the direction of the beam, the *x* axis is perpendicular to the beam in the horizontal plane pointing left as seen from the source, and the *y* axis points upwards (Willendrup *et al.*, 2024 ▸). In *BornAgain*, however, the average sample surface defines the *xy* plane, which is always referred to as ‘horizontal’, regardless of the orientation of the sample in laboratory space, and the mean incident beam lies in the *xz* plane, originating from the quadrant *x* < 0, *z* > 0 (Pospelov *et al.*, 2020 ▸).

Following the suggestion of placing the MCPL_output*McStas* component at the sample position, the neutron parameters in the MCPL file are already expressed relative to a sample at the origin, so the only task is to apply a coordinate rotation so that the parameters are expressed in accordance with the *BornAgain* conventions. The necessary coordinate rotation is calculated by the script based on the orientation of the sample (horizontal or vertical) and the intended incident angle of the beam.

The parameter actually used by the *BornAgain* API is α_i_ (the incident angle of the neutrons on the sample), and the output is a grid of outgoing angles (ϕ_i_ and ϕ_f_); hence, the labeling of the axes is irrelevant from the perspective of *BornAgain*, provided the values are handled correctly.

After the coordinate transformation, it is necessary to propagate the neutrons to the sample surface, because in *McStas* neutrons are only propagated to the last component before the MCPL_output component, while *BornAgain* makes calculations based on an input incident angle, without regard for the actual position of a neutron. By default, neutrons that would miss a sample of given dimensions are discarded before the *BornAgain* simulation, but this can be changed to direct propagation of them to the detector surface to allow over-illumination of the sample, or even a direct-beam simulation with no sample.

**Figure 3:**
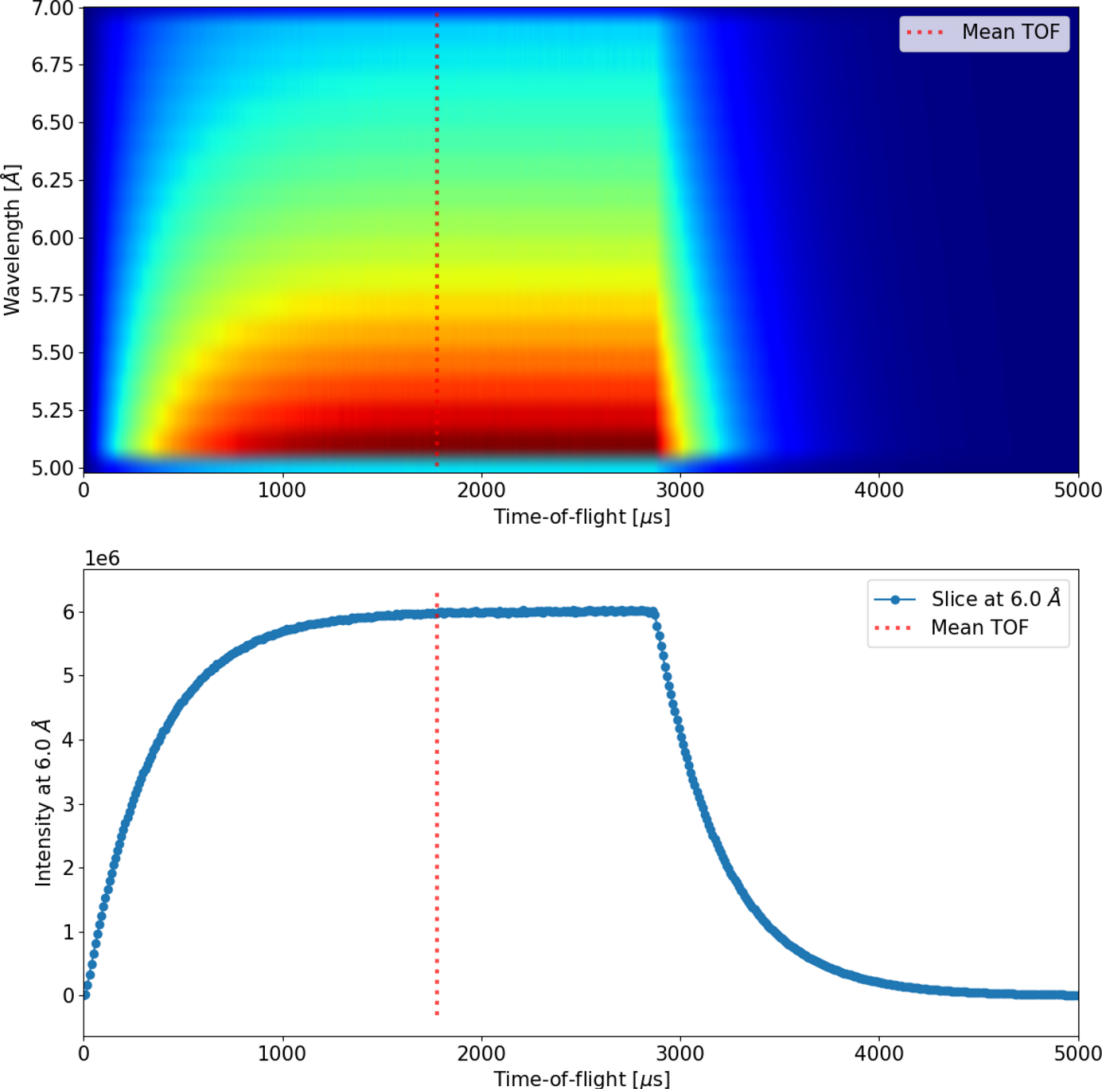
Demonstration of defining *t*_0_ automatically via a TOF–wavelength *McStas* monitor at the source position (top). The relevant wavelength bin containing the wavelength of interest (6.0 Å) is retrieved and the mean value is defined as the weighted average (bottom).

As an additional step during preconditioning for TOF instruments, an optional *t*_0_ correction can be applied to adjust the TOF values of the neutrons. The TOF value is not used in the *BornAgain* calculations; therefore, this correction is only required to ensure correct data reduction and could, in principle, be applied at a later stage. The *t*_0_ correction is intended to ensure that the simulated TOF of neutrons corresponds to the correct reference time by adjusting each TOF value by a certain *t*_0_ value. The value of *t*_0_ can be either provided by hand or calculated automatically using a TOF–wavelength *McStas* monitor placed at the reference position from where the TOF and flight path are to be calculated. For the latter case, as demonstrated in Fig. 3 ▸, *t*_0_ is calculated as the mean value (weighted average) of the TOF spectrum for the wavelength bin containing the wavelength of interest. In the example shown, the ESS_butterfly*McStas* source component is used, and the TOF–wavelength *McStas* monitor is placed at the same position. Without applying a *t*_0_ correction, the TOF of all neutrons would be overestimated by their initial emission time at the source position. By subtracting a *t*_0_ value from the TOF of all neutrons, the reference point of the TOF is shifted from the beginning of the pulse by that amount. When using a mean value of the initial time at the source position, roughly half of the neutrons will have their TOF slightly underestimated, and the other half slightly overestimated. This approach incorporates the uncertainty of the TOF introduced by the pulse width, just as expected in a real TOF measurement.

By default, during data reduction, the wavelength is calculated from the TOF and the flight path from the neutron source in the simulation. If the reference position from which the TOF and flight path are defined differs from the source position, both the TOF and the flight distance must be corrected. Such corrections are required for certain chopper modes, where the *t*_0_ correction becomes essential. One notable example is the WFM mode, which is intended to be used for some instruments at the European Spallation Source (ESS) (Löhmann *et al.*, 2020 ▸).

#### 2.2.3. Simulation

After the preconditioning, all neutrons are at the sample surface. A *BornAgain* sample model is loaded at this stage, which is used for neutron–sample interaction calculations through the *BornAgain* Python API that enables users to run simulations through Python scripts.

For each neutron – representing multiple or fractional real neutrons with identical properties – a *BornAgain* simulation is set up and run separately. In each *BornAgain* simulation, the beam is defined according to the parameters of the corresponding neutron, with the beam intensity, wavelength and incident angle determined by the neutron’s statistical weight, wavelength and velocity vector, respectively. For every case, a *BornAgain*SphericalDetector is configured with the desired angular coverage and number of pixels (number of outgoing directions). *BornAgain* assigns the center of each detector pixel as the direction of the outgoing scattering vector, so using the same SphericalDetector definition would mean that the outgoing directions are the same for each neutron. In order to better sample the scattering-direction space, an additional randomization is introduced, shifting the outgoing direction array from pointing to the center of the pixels to a random point within the pixels, thus producing a unique set of scattering directions for every simulated neutron. This random sampling approach is essential because in *BornAgain*, by default, the DWBA cross section is only evaluated at the center of the pixels (Pospelov *et al.*, 2020 ▸). This differential scattering cross section is then normalized by the solid angle covered by each pixel in order to get the probabilities of scattering toward each pixel. By introducing random variations within each pixel’s solid angle, the simulations stochastically sample the entire scattering-direction space, and the complete differential scattering cross section is approached in the limit of infinite incoming neutron rays. Importantly, the scaling is preserved as the number of outgoing directions (number of SphericalDetector pixels) is increased, because *BornAgain* will see smaller pixels and normalize accordingly. This SphericalDetector object is used only for the *BornAgain* simulation part of the workflow to define the outgoing directions and solid angles in the scattering calculations. In later processing steps, the detector surface defined by the user to represent the real detector is used instead.

After the *BornAgain* simulation, all outgoing rays of each neutron are propagated to the detector surface, while taking gravity into account, where scattering vector (**Q**) values are calculated with the following formula: 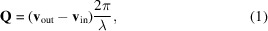
where **v**_out_ and **v**_in_ are the incident and outgoing direction vectors, and λ is the wavelength of the neutrons. The intent is to acquire **Q** values that could be derived from a real measurement, rather than getting exact values from parameters only known in simulations; therefore, the following considerations are made during the **Q** calculations to represent real data reduction. The starting point of the outgoing direction vector is the center of the sample surface, not the actual point of scattering. The end point of the outgoing direction vector is the center of the detector pixel the neutron enters, not the actual intersection of the outgoing neutron with the detector surface. Optionally, the pixel in which the neutron enters can be determined after applying a Gaussian smearing to the intersection point coordinates, to account for the statistical nature of the neutron detection process (*e.g.* local scattering, ionization along the path of conversion products, charge drift and electronic readout). The incident direction vector is universally calculated from the intended incident angle on the sample, not the individual incident direction of each neutron. The wavelength is calculated from the total TOF until the detector and the total flight path, where the latter is the sum of the nominal source-to-sample distance, provided as a fixed parameter, and the calculated sample-to-detector path length. The sample-to-detector path length is the calculated distance between the center of the sample surface and the center of the pixel the neutron enters. In the case of the WFM mode, the flight path of the neutron is reduced by the provided virtual source distance parameter, as its *t*_0_-corrected TOF value corresponds to that starting point instead of the source of neutrons in the simulation. In the case of single-wavelength instruments, the calculations are done with a fixed wavelength value selected by the monochromator.

The most time-consuming part of the script is the *BornAgain* simulation, due to the DWBA calculations. This step and the subsequent calculation of *Q* values are performed completely independently for each neutron. This independence presents an easy way for parallelization by dividing the incident neutron among CPU cores. The default option is to use parallelization with all available CPU cores minus two, but it can be altered or turned off completely using input options.

#### 2.2.4. Output

The result of the simulation and **Q** calculation is a list of **Q** events (weight, *Q*_*x*_, *Q*_*y*_, *Q*_*z*_), with the sum weights representing the number of neutrons per second. Due to the potentially large amount of data as a result of the combination of many incident neutrons from *McStas*, and many outgoing directions in the *BornAgain* simulation, the results can be histogrammed before being saved. The ranges covered by the histograms along each axis and the number of bins in each direction can be controlled by input values, which will define how large the output file will be, regardless of the number of simulated neutrons. Uncertainties of the bin values are also saved, estimated by the square root of the summed weight squares, as is common practice in Monte Carlo simulations, *e.g.* in *McStas* (Willendrup *et al.*, 2024 ▸).

At the end of the *BornAgain* simulation script run, the histogrammed results are saved in the .npz file format of *NumPy* (https://www.numpy.org/), facilitating easy processing with Python scripting. Optionally, a basic 2D *Q* plot can be created that is intended for diagnostic checks of results of low statistics simulations, but for proper data processing and plotting, the dedicated script provided in the package, or custom ones, should be used.

### 2.3. Data processing and visualization

The package includes a script dedicated to plotting *Q* values derived from the simulation results, offering options for creating 1D and 2D plots, comparing multiple datasets, and adjusting plot parameters. It is hardly possible to cover all individual data-processing and visualization needs by one solution; therefore, the script is modularized to facilitate the implementation of custom solutions by the users, for which this script can serve as a blueprint.

A key feature of this script is the option to upscale the simulation results to a certain experiment time in order to make the results comparable to real measured data. In most cases, *McStas* neutron sources are defined to provide fluxes normalized to one second; therefore, all *Q* plots created from the simulation results usually correspond to a one second ‘virtual experiment’ time. For simulating actual experiments, the intensities are scaled by the given counting time and Poisson statistics are applied to obtain realistic neutron counts. To facilitate the comparison with measured data, a hard-coded example of loading data from NeXus files is available.

In order to achieve better agreement with the measured data, an option to add Poisson background to the simulated data is provided. This step is purely cosmetic and does not correspond to any underlying physical process, serving only for visualization purposes.

## 3. Example usage

In order to verify the framework, we compare our *mcstas_gisans* simulations with measured GISANS data. The source of the data is a structural characterization of nanoparticle-supported lipid bilayer arrays by grazing-incidence X-ray and neutron scattering (Paracini *et al.*, 2023 ▸). The samples are a monolayer of non-porous monodisperse silicon oxide spheres of a nominal diameter of 100 nm on a silicon substrate, measured in air. The GISANS measurements were performed at the ILL on the SANS instrument D22 (Paracini *et al.*, 2021 ▸). For detailed sample preparation and experimental setup, the reader is referred to Paracini *et al.* (2023 ▸) and the supporting information therein.

**Figure 4:**
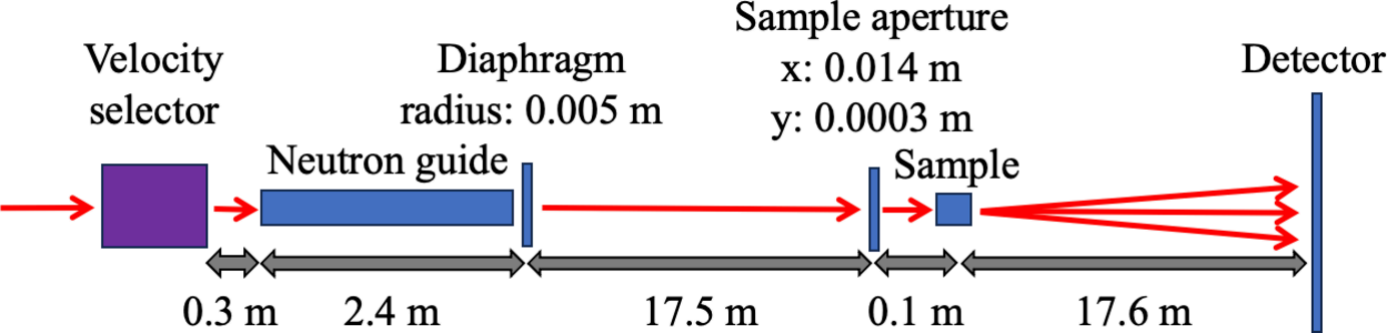
Schematic model of the D22 experimental setup recreated in *McStas*.

The *McStas* modeling of the D22 instrument was based on the ILL_H512_D22.instr instrument file from the *McStas* instrument examples library. It was extended by a Diaphragm and a Slit component for the collimation, TOFLambda_monitor components for the source and sample positions, and an MCPL_output component at the sample position to fit the requirements of the framework. The distances and instrument parameters were set in accordance with the experimental setup, illustrated in Fig. 4 ▸.

The *BornAgain* sample model used in the GISAXS simulations (Paracini *et al.*, 2023 ▸) was adapted for GISANS simulation by changing the scattering length density of the materials. Otherwise the model was kept unchanged, consisting of a layer of silicon oxide spheres in a finite 2D hexagonal lattice on a silicon oxide layer supported by a silicon substrate. The finite 2D lattice of variable size was defined by a silica radius parameter and the distance between the spheres given by a lattice parameter. The unit cells of the finite lattices were averaged over the lattice rotation angle χ, *i.e.* averaging the signal for all possible in-plane orientations of the hexagonal unit cells.

Due to imperfect modeling, the D22 *McStas* simulation yielded a higher intensity at the sample position than was expected from the measurements. To correct for this, an intensity scaling factor was introduced. The factor was determined by simulating the direct-beam measurement and calculating the ratio between the detected intensities in the measurement and the simulation. This ratio was then used to reduce the statistical weight of all neutrons in the *BornAgain* simulation script for simulations including samples. This normalization kept all other beam properties unchanged and also compensated for the lack of detection efficiency in the simulations, making the simulated results directly comparable with the measured values.

**Figure 5:**
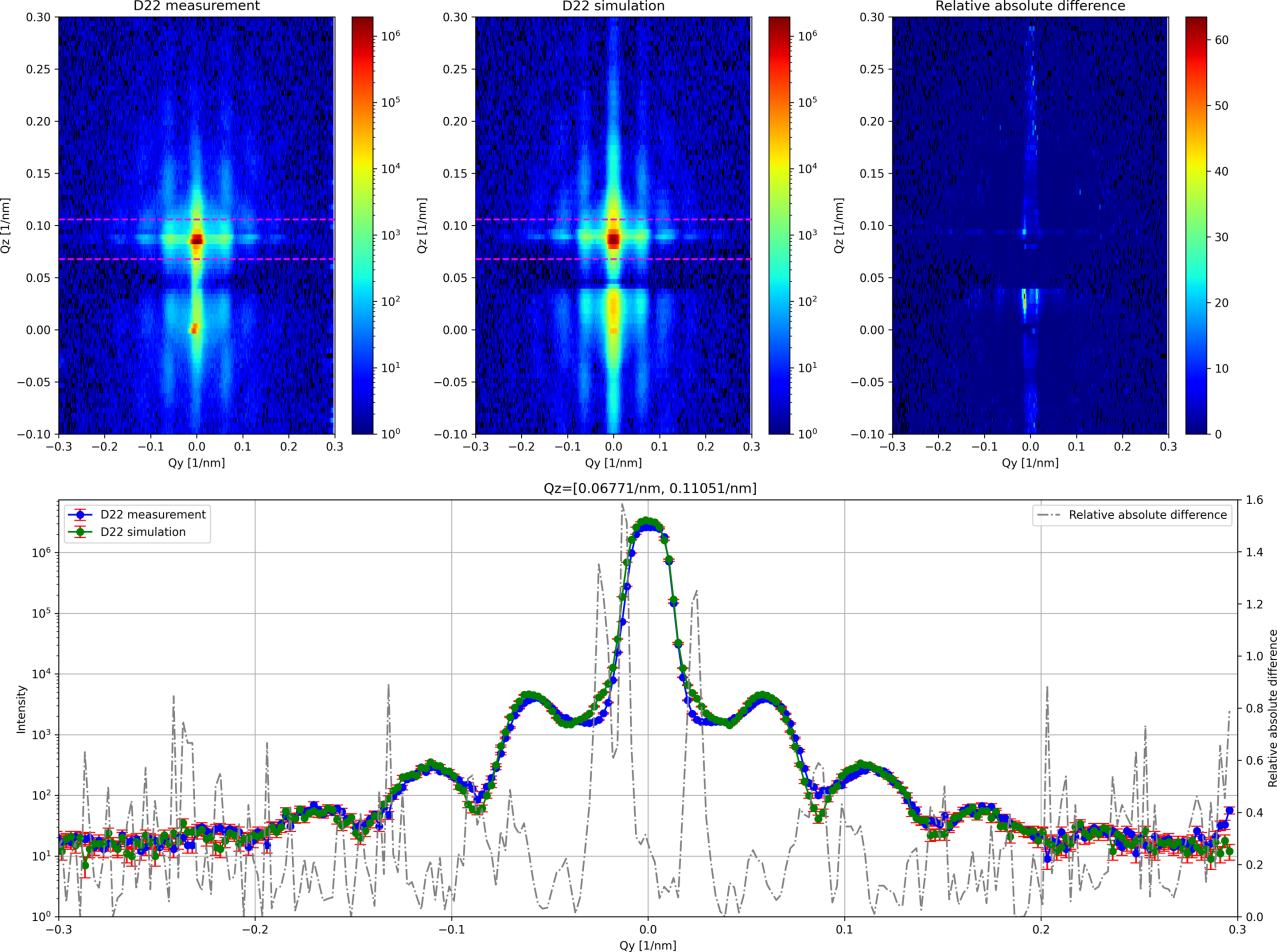
Comparison of measured and simulated GISANS data, visualized with the plotting script of the package. The figures in the top row show the 2D *Q* plots from the measurement (left) and the simulation (middle), and their relative absolute difference defined as |measurement − simulation|/measurement (right). The dashed lines indicate the regions for which the integrated 1D *Q* curves are depicted in the figure on the bottom, where the relative absolute difference for each data point is also shown on a secondary *y* axis. The limits of integration are also indicated over the bottom figure.

Through systematic manual variation of the sample parameters within reasonable ranges, the best match between simulation and experimental data was found for a silica sphere radius of 51 nm, a lattice size of 5 × 5 spheres, and a lattice parameter of 114 nm. After adding a Poisson background to the simulated data, the agreement with the measured data is convincing, as depicted in Fig. 5 ▸. The parameters obtained from the GISANS measurement and simulation are in good agreement and the best match was found for a silica sphere radius of 53 nm, a lattice size of 5 × 5 spheres, and a lattice parameter of 110 nm, as shown in Table S2 and Fig. S4 of the supporting information for Paracini *et al.* (2023 ▸). The presented level of agreement was achieved without automated parameter optimization. Significant deviations between simulation and experiment can be observed in certain regions, most notably around the specular peak. With more elaborate modeling and data analysis, the numerical agreement could be improved; however, this comparison is intended as a validation of the simulation framework rather than as a fully optimized fit to the experimental data. The exact versions of the simulation software used in this work are *mcstas_gisans* 2.0, *McStas* 3.5.16 and *BornAgain* 21.2.

The *mcstas_gisans* package has also been used in a study presenting a focusing nested mirror optics solution named PortGISANS that can improve the GISANS capabilities of SANS beamlines (Mehler *et al.*, 2025 ▸). In that work, it was used as a tool to predict and benchmark the performance of the nested mirror assembly by combining *McStas* and *BornAgain* simulations through this framework.

## 4. Limitations and approximations

The proposed framework is in an early stage of development and is therefore subject to a number of limitations and approximations. Some of these stem from the lack of need from current use cases, and are expected to be resolved in the future. Suggestions and improvements are welcome via the GitHub repository, either through the issue tracker or by submitting a pull request.

TOF filtering is based on TOF at the sample position, instead of that at the detector surface, which would be the correct solution. The reason for this approximation is that the TOF at the sample position is known from the *McStas* simulation, as opposed to the TOF at the detector surface, which would require performing the *BornAgain* simulation of neutrons that could then be filtered out. Given that the most time-consuming part is the *McStas* simulation, this could be changed, and with further development and powerful computers, it could be possible to simulate multiple TOF/wavelength bins together and produce the results for all of them simultaneously.

The most notable sample-related limitation is the lack of support for magnetic materials. This is simply due to the lack of polarization handling in the *mcstas_gisans* codebase. *McStas*, MCPL and *BornAgain* are all capable of handling polarization, so this could be changed later on. Similarly, a purely technical limitation concerns the treatment of azimuthal incidence: the azimuthal angle of the incoming neutrons is currently implemented as an offset to the outgoing angles, while the scattering interaction itself is always evaluated at the default value ϕ = 0. This restriction is likewise not fundamental and could be resolved with minor code modifications.

Most of the limitations and approximations are tied to the detectors. The most prominent limitation is the lack of simulation of the detection process, which practically means that the defined detector surface has a uniform efficiency of 100% without any positional or neutron-energy dependence. Also, the propagation of the neutrons to the detector surface is based on the assumption that the detector surface is vertical in the laboratory frame.

The inbuilt options to add Poisson or constant background to the simulations in *BornAgain* cannot be used within this framework. Instead, it can be done later at the data-processing and visualization step, where a Poisson background of chosen expected count value can be added.

Generally, modeling of the beam transport through the instrument and the GISANS processes at the sample can only be as good as modeling of the respective parts in *McStas* and *BornAgain*. One known issue for *BornAgain* is that, when choosing to include the specular reflected beam intensity along with the scattered intensity in a grazing-incidence small-angle scattering simulation, the scattered-intensity loss is not accounted for in the intensity of the specular beam. This results in a slightly overestimated specular peak intensity (Wuttke, 2021 ▸), which is usually not a concern for typical *BornAgain* applications.

The *mcstas_gisans* package is currently only tested with *BornAgain* versions 21.1 and 21.2. It is likely to work with other versions as well, with suitable sample models, but depending on the operating system, setting up the required software environment might differ from the suggested way described later in Section 5.

## 5. Distribution and usage

The codebase of *mcstas_gisans* is publicly accessible in a Git repository, which is currently hosted at https://github.com/MilanKlausz/mcstas_gisans, where a link to the detailed documentation is also available. The recommended way of using the framework is cloning the repository to the computer where it is intended to be run and setting up a Conda (Ana­conda, 2017 ▸) environment using the provided conda.yml file. The Conda environment should install the package and cover all the dependencies, with the only exception being *McStas*, which is expected to be already installed. Nevertheless, users without working *McStas* installations could add *McStas* to the Conda environment file, or follow the *McStas* installation guide on the official website (https://www.mcstas.org/). After activating the created Conda environment, the user should have access to the main commands: mg_run for running the simulation and mg_plot for processing and plotting the results.

If the user wants to use a *McStas* instrument model already present in the resources/mcstas_models directory of the repository, and a *BornAgain* sample model built in the package, the steps of usage are (1) running the *McStas* simulation in a shell set up for running *McStas* or using the *McStas* graphical user interface, (2) running the *BornAgain* simulation script (mg_run) using the MCPL output file from the *McStas* simulation in a shell with the Conda environment activated, and (3) plotting the result with the plotting script (mg_plot) in a shell with the Conda environment activated.

If the user wants to use a new *McStas* instrument file, it should be prepared by adding the necessary components to it (MCPL_output at the sample position and optional monitor components), and the user should describe the instrument in the instruments.py module of the framework on the basis of the already present examples. This second step will ensure that the *BornAgain* simulation script will use correct parameters for the calculations and find the monitor outputs.

If the user wants to use a custom *BornAgain* sample model rather than one of the samples built into the package, it can be provided in a Python module format. Such a custom sample module can be created by hand or created and exported from the *BornAgain* GUI. Any new parametrized sample model is supported out of the box by an input parameter of the mg_run script, which accepts an arbitrary list of ‘argument = value’ pairs that are parsed and passed to the sample model.

The small-slit settings that are usual for GISANS measurements necessitate computationally heavy *McStas* simulations to yield a reasonable number of neutrons at the sample position. The user should ensure a stable simulated neutron intensity at the sample position by increasing the number of simulated source particles, or employing other variance-reduction techniques. This task can easily demand the computational power of a computer cluster. The *BornAgain* simulation part can also benefit from a strong computer or computer cluster. The driving factors there are the number of incident neutrons and simulated outgoing scattering directions. Lowering the number of outgoing directions can greatly reduce the computational effort, but it can cause artefacts around sharp peaks (*e.g.* the specular peak). The stability of the results should also be checked by the user by altering this parameter.

## 6. Conclusions

To optimize instrumentation for GISANS, we have developed *mcstas_gisans*, a framework that bridges the gap between existing simulation tools. One particular use for this tool will be aiding the comparison of the performance of new and existing neutron scattering instruments with respect to GISANS data collection. This is of particular use to support the design of dedicated GISANS instrumentation, such as the SAGA project at the ESS, the project ongoing at the High Brilliance Source in Germany and the project for a dedicated GISANS instrument at the Paul Scherrer Institute in Switzerland. The present work shows that our software tool is able to correctly calculate GISANS scattering patterns by combining *McStas* and *BornAgain* simulations. The scattering from a 2D crystal of silica spheres deposited on a silicon surface measured at D22 (ILL) is correctly calculated. This opens up the possibility to predict instrument performance for GISANS and to support design choices for instruments as well as instrument upgrades. Furthermore, it enables the optimization of samples and experimental setups by providing absolute intensities with realistic statistical uncertainties. As a yet untested experimental option, simulation with photons is also supported by our framework, making it possible to use it for GISAXS simulation by the connection of an X-ray simulation code (*e.g.McXtrace*) with *BornAgain*.

We are working to replace the described workflow with a more integrated and user-friendly framework (https://github.com/aglavic/mcstas_gisans). In the new implementation, the *BornAgain* calculation via a Python interface is integrated into the *McStas* model by a custom component that can be placed directly like other samples. This coupling is achieved by running a *BornAgain* simulation service that communicates directly with *McStas* during runtime. The dedicated component in *McStas* sends neutron events to the service for scattering calculations and receives the resulting outgoing rays for continued propagation within *McStas*. This approach will enable the use of standard *McStas* detector and monitor components for data collection, eliminate the need for intermediate data files, and simplify the overall workflow. The processing and visualization of detector outputs will remain as a separate flexible step for data analysis, the same way as for other instrument models. Although *McStas* code provides facilities to directly link a C or C++ library to an instrument simulation, in the case of *BornAgain*, the potential performance benefits of such a direct connection in *McStas* would only be marginal for real-world examples. With the provided Python interface, existing *BornAgain* models can be used without additional effort.

## Data Availability

The data and code used to generate the results in this paper are available in the data/paper/ and examples/paper/ directories of the *mcstas_gisans* GitHub repository at https://github.com/MilanKlausz/mcstas_gisans.
